# A Peculiar Device

**Published:** 1881-02

**Authors:** 


A Peculiar Device to terminate the chloroform narcosis is mentioned by Schirmer in the February number the *Centralblatt f. Augenheilkunde.* He claims to have used it in his clinic for many years, and often succeeded in producing inspiratory movements when other means failed. He also employed it to induce rapid recovery, for instance, in strabismus operations, in order to test the result. The method consists in irritating the nasal mucous membrane. It has long been known, at least to physiologists, that the fifth nerve retains its sensibility longer than any part in narcosis, and that reflexes may be induced through this nerve when other irritations fail. Schirmer uses simply a roll d piece of paper, which he turns in the nose. In dangerous cases he dips the paper into ammonia.—*Medical Press and Circular.*

